# Conversion surgery for microsatellite instability-high gastric cancer with a complete pathological response to pembrolizumab: a case report

**DOI:** 10.1186/s12957-022-02661-8

**Published:** 2022-06-10

**Authors:** Yoshifumi Hidaka, Takaaki Arigami, Yusaku Osako, Ryosuke Desaki, Masahiro Hamanoue, Sonshin Takao, Mari Kirishima, Takao Ohtsuka

**Affiliations:** 1 Department of Surgery, Tanegashima Medical Center, 7463 Nishinoomote, Nishinoomote, Kagoshima 891-3198 Japan; 2 Department of Onco-biological Surgery, Kagoshima University Graduate School of Medical and Dental Sciences, 9-35-1 Sakuragaoka, Kagoshima, 890-8520 Japan; 3 Department of Pathology, Field of Oncology, Graduate School of Medical and Dental Sciences, Kagoshima University, 8-35-1 Sakuragaoka, Kagoshima, 890-8520 Japan; 4 Department of Digestive Surgery, Breast and Thyroid Surgery, Graduate School of Medical and Dental Science, Kagoshima University, 8-35-1 Sakuragaoka, Kagoshima, 890-8520 Japan

**Keywords:** Unresectable gastric cancer, MSI-high, Pembrolizumab, Conversion surgery, R0 resection

## Abstract

**Background:**

Immune checkpoint inhibitors are reportedly effective in treating microsatellite instability (MSI)-high gastric cancer. There are a few case reports of conversion surgery (CS) with nivolumab but none with pembrolizumab. Herein, we describe a patient with MSI-high gastric cancer who was successfully treated with pembrolizumab and underwent CS with a pathological complete response.

**Case presentation:**

A 69-year-old man was diagnosed with stage III gastric cancer (T3N2M0) based on contrast-enhanced computed tomography, which revealed a neoplastic lesion and enlarged perigastric lymph nodes in the gastric lesser curvature. The anterior superior lymph node of the common hepatic artery (CHA) was determined to be unresectable due to invasion of the pancreatic head and CHA. Histopathologically, the biopsied tissue showed moderately differentiated adenocarcinoma, then determined to be MSI-high. After three courses of mFOLFOX6 therapy, the patient was diagnosed with progressive disease. Since one course of paclitaxel plus ramucirumab therapy caused grade 3 fatigue, his second-line therapy was switched to pembrolizumab monotherapy. After three courses, the primary tumor and perigastric lymph nodes had shrunk, and it was determined as a partial response. The anterior superior lymph node of the CHA became resectable based on the improvement of infiltration of the pancreatic head and CHA due to shrinkage of the lymph node. Tumor markers remained low; hence, distal gastrectomy plus D2 lymphadenectomy was performed at the end of six courses. Anterior superior lymph node of the CHA was confirmed by intraoperative ultrasonography, and the resection was completed safely. The gross examination of the resected specimen revealed an ulcer scar at the primary tumor site. The histopathological examination showed no viable tumor cell remnants in the primary tumor, which had a grade 3 histological response, and resection margins were negative. The lymph nodes showed mucus retention only in the anterior superior lymph node of the CHA, indicating the presence of metastasis, but no viable tumor cells remained. The patient commenced 6 months of adjuvant pembrolizumab monotherapy 3 months after surgery. Twenty months after surgery, there was no evidence of recurrence.

**Conclusions:**

Conversion surgery following pembrolizumab monotherapy has a potential utility for the treatment of MSI-high gastric cancer.

## Background

The conventional treatment of gastric cancer is based on the histological classification of pathological findings, and treatment decisions are determined based on the degree of wall invasion, lymph node metastasis, and presence or absence of distant metastasis. The application of a precision medicine in the treatment of gastric cancer began in 2010 when the Trastuzumab for Gastric Cancer study demonstrated the efficacy of trastuzumab in human epidermal growth factor receptor 2 (HER2)-positive gastric cancer [1]. Additionally, further advances in the application of precision medicine in gastric cancer have been possible due to the results of exome analysis, DNA methylation array analysis, and chromosome copy number analysis of gastric cancer from the 2014 cancer genome atlas project, which identified four major molecular subtypes of gastric cancer (the Epstein-Barr virus subtype, microsatellite instability subtype, the genomically stable subtype, and the chromosomal instability subtype) [2].

Of these, microsatellite instability (MSI) tumors accounting for 22% demonstrate frequent genomic alteration in microsatellites induced by mismatch repair deficiency (dMMR) [3]. Accumulated mutational burden in such tumors leads to the upregulation of immune checkpoint proteins: immune cells exhibit programmed death 1 (PD-1) and MSI tumor cells exhibit programmed death-ligand 1 (PD-L1) [4]. PD-1/PD-L1 pathway induces immune tolerance, enabling tumor cells to survive. Immune checkpoint inhibitors (ICIs) show high-therapeutic efficacy against MSI-high/dMMR/PD-L1-positive tumor cells by inhibiting the binding between PD-1 and PD-L1, preventing immune cell suppression [5].

In Japan, the recommended second- and third-line PD-1 inhibitors for unresectable or recurrent gastric cancer in MSI-high patients are pembrolizumab and nivolumab, respectively. There have been a few reports of conversion surgery (CS) with nivolumab, but none with pembrolizumab to date [6–12]. We report a case of MSI-high gastric cancer in which pembrolizumab treatment was successful, CS was performed, and a pathological complete response was obtained.

## Case presentation

**Fig. 1:**
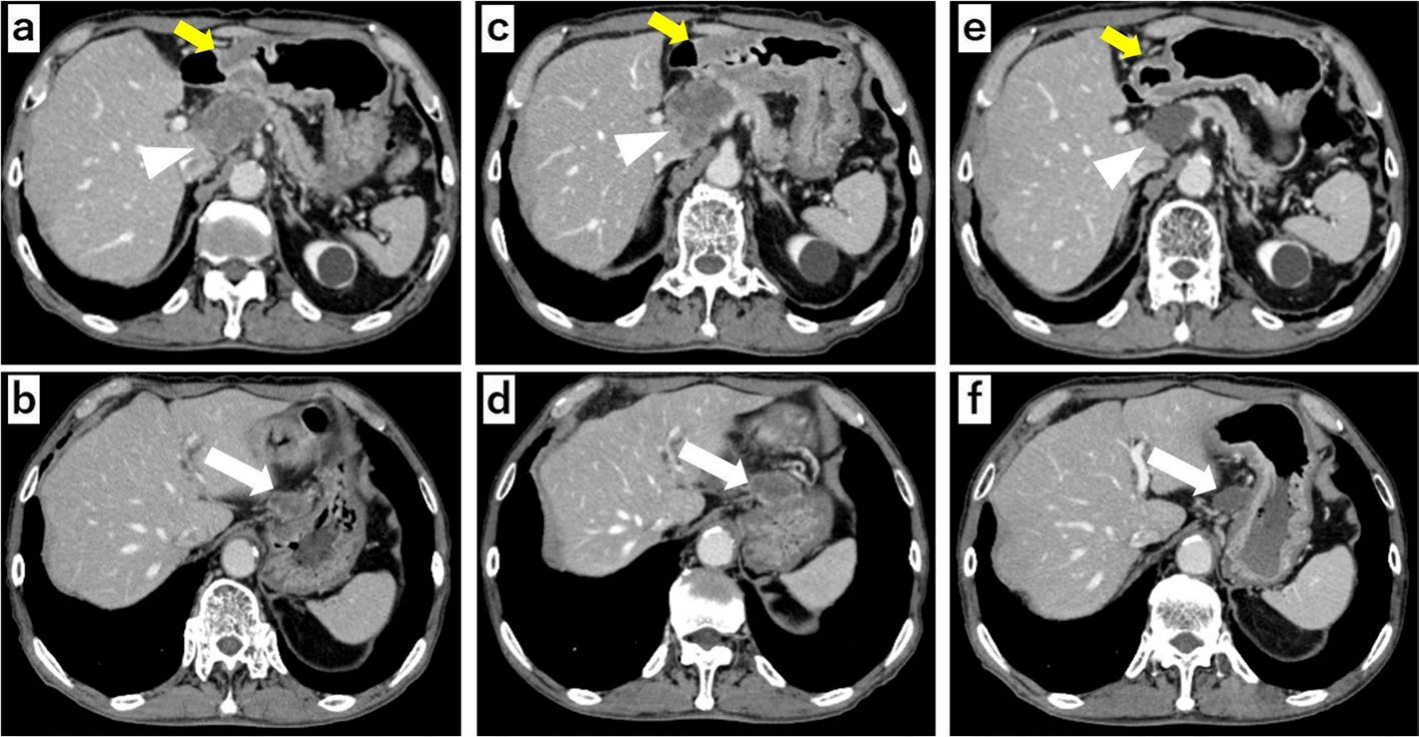
Contrast-enhanced CT scan. **a**, **b** Before chemotherapy (yellow arrows: primary tumor, arrowheads: anterior superior lymph nodes of the common hepatic artery, white arrows: lesser curvature lymph nodes). **c**, **d** After three courses of mFOLFOX6. **e**, **f** After three courses of pembrolizumab

**Fig. 2:**
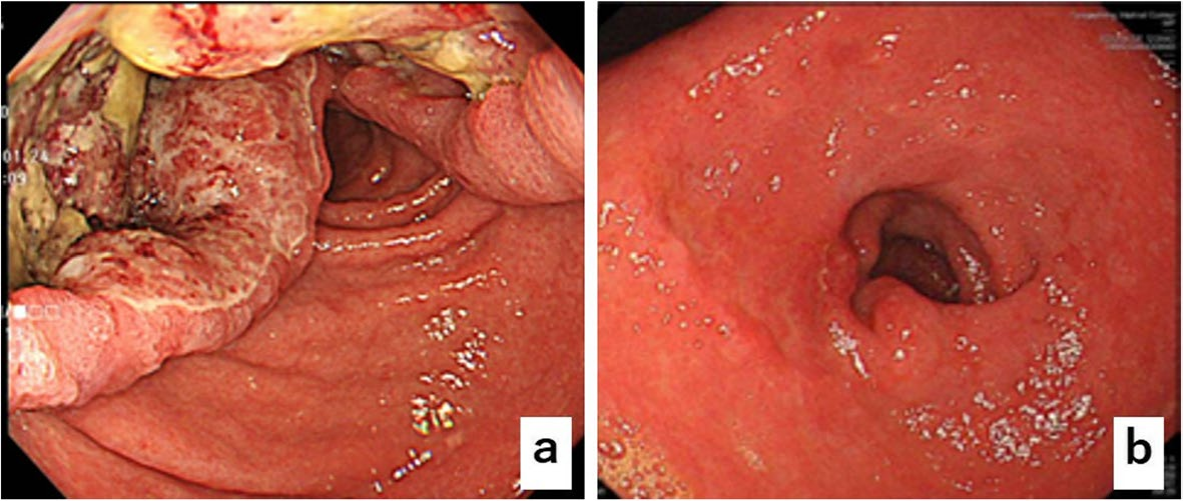
**a** Esophagogastroduodenoscopy reveals a tumor with an ulcer on the lesser curvature of lower body of the stomach. **b** Tumor in the primary lesion almost disappears after six courses of pembrolizumab

The patient was a 69-year-old man referred to our department after a simple computed tomography (CT) scan revealed enlarged gastric lesser curvature lymph nodes and a mass lesion in the pancreatic head. A subsequent contrast-enhanced CT revealed wall thickening of the lower gastric body, multiple enlarged gastric lesser curvature lymph nodes, and an anterior superior common hepatic artery (CHA) lymph node involving the pancreatic head and CHA (Fig. 1a, b). Tumor markers showed elevated levels of carcinoembryonic antigen (CEA, 33.9 ng/ml) and carbohydrate antigen (CA19-9, 108.1 U/ml). Esophagogastroduodenoscopy (EGD) revealed a tumor with an ulcer on the lesser curvature of the lower body of the stomach (Fig. 2a). Histopathological examination of the biopsied specimen indicated moderately differentiated adenocarcinoma, which was HER2-negative, and MSI-high status was determined using an MSI test kit (FALCO biosystems, Kyoto, Japan). Based on these findings, a diagnosis of advanced gastric cancer of stage III (T3N2M0) was made (8th edition of the Union for International Cancer Control). The anterior superior lymph node of the CHA was considered unresectable due to its involvement with the pancreatic head and CHA, and chemotherapy was decided as the treatment plan. The patient had difficulty swallowing at the time of examination; therefore, he was treated with mFOLFOX6 therapy (leucovorin 200 mg/m^2^, oxaliplatin 85 mg/m^2^ in a 2-h infusion, bolus fluorouracil 400 mg/m^2^ on day 1, and a 46-h infusion of fluorouracil 2400 mg/m^2^ every 2 weeks). At the end of three courses, tumor markers were elevated (CEA, 35.3 ng/ml and CA19-9, 136.9 U/ml), and contrast-enhanced CT showed enlargement of the primary tumor, and lymph node of the lesser curvature and anterior superior CHA (Fig. 1c, d).

**Fig. 3:**
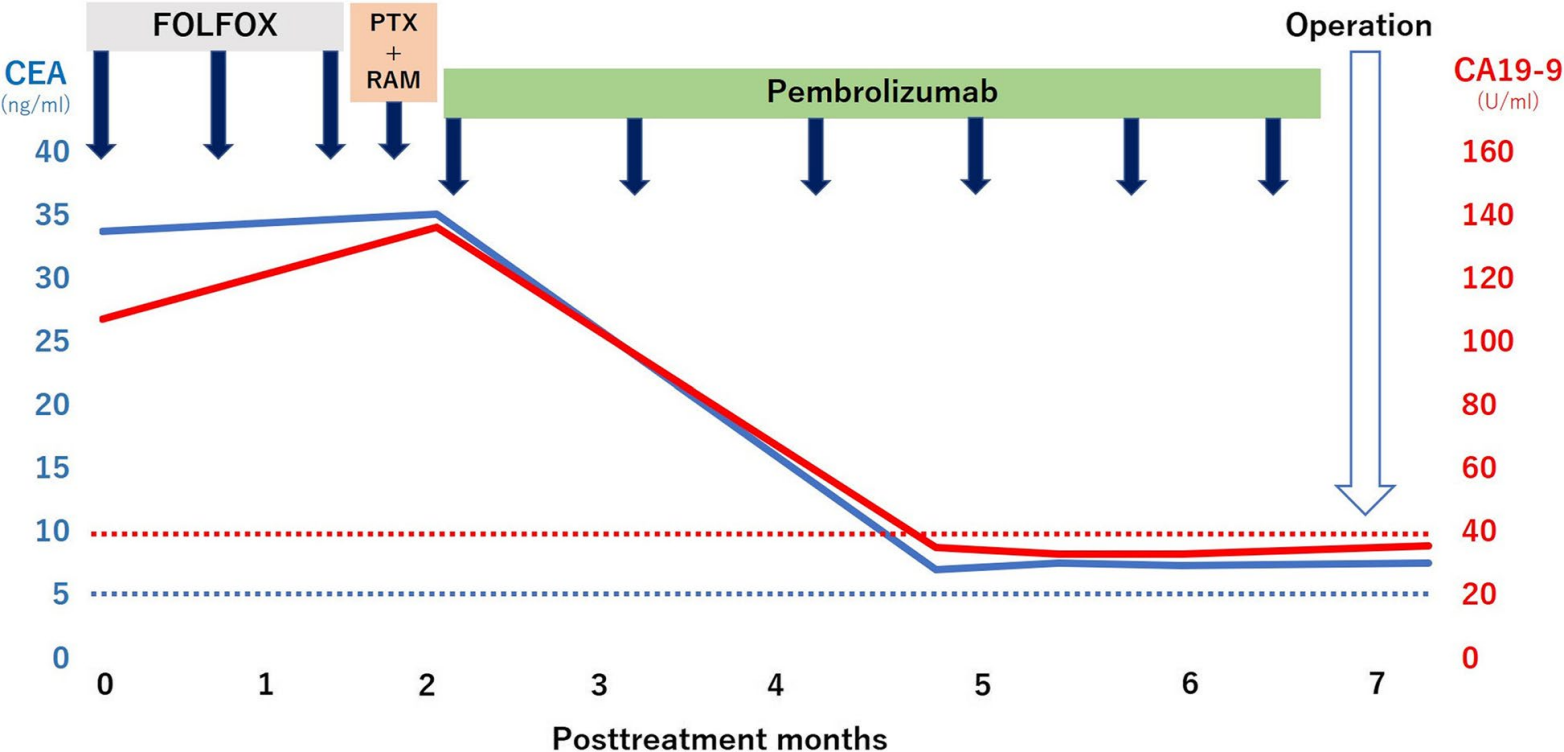
After three courses of pembrolizumab, both CEA and CA19-9 values decrease

**Fig. 4:**
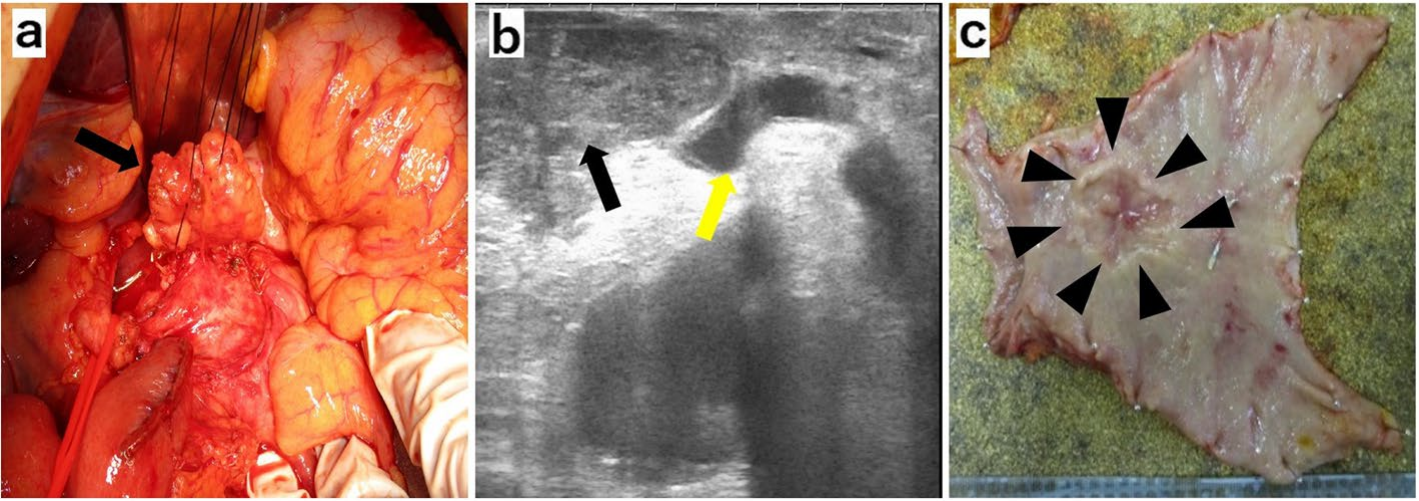
**a** Intraoperative photographs of the anterior superior common hepatic artery lymph node (black arrow). **b** Intraoperative ultrasonography image shows no infiltration of the anterior superior lymph node of the common hepatic artery (black arrow) into the common hepatic artery (yellow arrow). **c** Macroscopic findings of the resected stomach. An ulcer scar is seen in the primary tumor site (arrowhead)

**Fig. 5:**
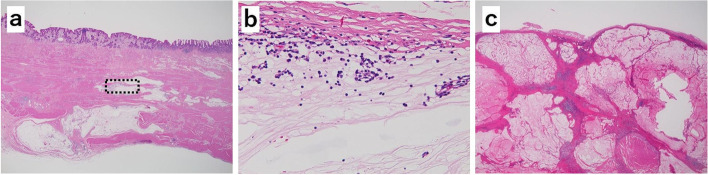
Histopathological findings (hematoxylin-eosin staining). **a** Primary gastric lesion (×40 magnification). Mucus accumulation is observed from the submucosal layer to the subserosal layer. **b** Primary gastric lesion (×400 magnification). Infiltration of lymphocytes in the mucus, and no viable residue of tumor cells is observed. **c** Metastatic lesions of the anterior superior lymph node of the common hepatic artery. Mucus accumulation without viable residue of tumor cells is found in the lymph nodes

The patient was diagnosed with the progressive disease based on the Response Evaluation Criteria in Solid Tumors (RECIST) and was treated with ramucirumab plus paclitaxel as a second-line therapy. After a 4-week course of paclitaxel (80 mg/m^2^) intravenously on days 1, 8, and 15, with ramucirumab (8 mg/kg) intravenously on days 1 and 15, the patient developed a grade 3 fatigue, which necessitated a change of the therapeutic drugs. Hence, his second-line therapy was switched to pembrolizumab monotherapy (pembrolizumab 200 mg once every 3 weeks). Tumor markers decreased markedly to CEA 7.2 ng/ml and CA19-9 35.9 U/ml at the end of three courses, and contrast-enhanced CT showed that both the primary tumor and the lesser curvature lymph node had shrunk, and the anterior superior lymph nodes of the CHA showed improved findings of invasion into the pancreatic head and CHA (Fig. 1e, f). A diagnosis of partial response was made based on RECIST guidelines. EGD revealed a tumor in the primary lesion that almost disappeared after six courses of pembrolizumab (Fig. 2b). Tumor markers remained low with CEA 7.5 mg/ml and CA19-9 33.7 U/ml (Fig. 3), and contrast-enhanced CT revealed that R0 resection was possible; therefore, a distal gastrectomy with D2 lymph node dissection was performed. Surgical findings showed no obvious liver metastasis or peritoneal dissemination, and the cytology was negative. The anterior superior lymph node of the CHA was safely dissected after resection of the primary tumor, as intraoperative ultrasonography confirmed that there was no invasion into the pancreatic head or CHA (Fig. 4a, b). Gastrectomy specimens showed an ulcerated scar on gross examination (Fig. 4c). The histopathological findings showed that the superficial layer of the gastric mucosa was covered by regenerating epithelium and that there was only mucus accumulation from the submucosa to the serosa with no residual tumor cells that could be considered viable, determined to be histological grade 3 (Fig. 5a, b). The histopathological findings of the resected lymph node revealed mucus accumulation only in the anterior superior lymph node of the CHA, which was diagnosed as metastasis of adenocarcinoma, although there were no residual tumor cells that could be classified as viable (Fig. 5c). The patient was discharged from the hospital 23 days after surgery without complications.

The patient commenced 6 months of adjuvant chemotherapy with pembrolizumab 3 months after surgery. Twenty months after surgery, the patient is alive and recurrence-free.

## Discussion

This is a case of a patient with unresectable advanced MSI-high gastric cancer who responded well to pembrolizumab, with a confirmed pathological complete response after CS. To our knowledge, this is the first report of such a case.

MSI-high gastric cancer has been reported to respond well to pembrolizumab and poorly to cytotoxic chemotherapy. KEYNOTE-061 compared pembrolizumab with paclitaxel in patients with advanced gastric or gastro-esophageal junction cancer that progressed with the first-line chemotherapy, and pembrolizumab showed better overall survival (OS) and objective response rate (ORR) than paclitaxel in a subgroup analysis of MSI-high/PD-L1-positive patients (median OS: not reached and 8.1 months; ORR: 46.7% and 16.7%, in pembrolizumab vs. paclitaxel groups; respectively) [13]. KEYNOTE-062 investigated the effect of pembrolizumab on chemotherapy (cisplatin + fluorouracil; CF) in patients with untreated gastric or gastro-esophageal junction cancer, but the combination failed to demonstrate OS superiority over chemotherapy (pembrolizumab + CF vs. CF, median OS: 12.5 vs. 11.1 months, respectively) [14]. KEYNOTE-158 evaluated the efficacy of pembrolizumab in MSI-high/dMMR cancer refractory to standard therapy, and the results were favorable, with an ORR of 45.8% in 24 patients with gastric cancer, including four complete response cases and seven partial response cases [15]. Based on these results, the Japanese Gastric Cancer Treatment Guidelines 2021 recommend pembrolizumab monotherapy as the second line in MSI-high gastric cancer. Furthermore, evidence has shown that cytotoxic chemotherapy does not improve and may even worsen the prognosis in MSI-high gastric cancer patients. In a retrospective analysis of postoperative stage II and stage III gastric cancer patients, the hazard ratio of MSI-high vs. MSI-low or microsatellite stable (MSS) tumors was 0.49 without chemotherapy. The hazard ratio decreased to 1.16 when adjuvant chemotherapy (5-fluorouracil only or 5-fluorouracil + platinum, anthracycline, taxane, etc.) was provided [16]. Smith et al. also reported that perioperative chemotherapy (epirubicin, cisplatin, and fluorouracil chemotherapy) was ineffective in MSI-high patients with gastric cancer [17]. In patients treated with surgery alone, OS was better for those with MSI-high tumors than those with MSI-low or MSS tumors (median OS: not reached vs. 20.3 months, in MSI-high vs. MSI-low or MSS tumors, respectively). In contrast, in patients treated with perioperative chemotherapy plus surgery, OS was better for those with MSI-low or MSS than for those with MSI-high tumors (median OS: 22.5 months vs. 9.6 months, for MSI-low or MSS tumors vs. MSI-high tumors). Hence, in patients with MSI-high gastric cancer refractory to cytotoxic chemotherapy, as in the present case, pembrolizumab monotherapy from the second line may render a better outcome with a more achievable chance for CS.

CS can be an effective treatment if chemotherapy is successful and R0 resection is possible. Since the REGATTA trial revealed that gastrectomy in patients with nonhealing factors (liver, peritoneal, or paraaortic lymph node metastases) did not improve OS (2 years in the chemotherapy alone vs. gastrectomy + postoperative chemotherapy group, OS: 31.7% and 25.1%, respectively) [18], chemotherapy is the standard treatment for gastric cancer with non-curative factors. However, with the recent advances in molecular classification of gastric cancer and the advent of chemotherapy with excellent antitumor effects, an increasing number of cases have been reported in which the non-curative factors have disappeared, and CS has improved the prognosis. A meta-analysis by Du et al. reported the efficacy of CS in unresectable advanced gastric cancer [19]. In the analysis of the CS vs. non-CS and R0 resection vs. non-R0 resection groups, 1-year and 3-year survival rates were superior in the CS and R0 resection groups, respectively. Morgagni et al. also reported that the CS group had a longer median survival time (MST) than the chemotherapy and best supportive care (BSC) groups (MST: 50 months, 14 months, and 3 months in the CS vs. chemotherapy vs. BSC groups, respectively) [20].

There are many case reports of CS by ICIs, in which curative resection was achieved with improvement or disappearance of non-curative factors using nivolumab in the third-line of stage IV gastric cancer [6–12]. In all case reports, R0 resection was performed by CS after confirmation of the disappearance of the lung [6] and liver [7] metastases, peritoneal dissemination [8, 9], and reduction of paraaortic lymph node metastases [10–12] by imaging findings such as contrast-enhanced CT, magnetic resonance imaging, and positron emission tomography-CT, and the patients did not experience postoperative recurrence. Beom et al. found that prognostic factors after CS for stage IV gastric cancer were complete macroscopic resection, chemotherapy response of metastatic sites (complete response/partial response), and change in CEA level [21].

However, there are no reports of CS with pembrolizumab to the best of our knowledge. In the present case, contrast-enhanced CT findings after pembrolizumab administration showed shrinkage of the metastatic lesions in the anterior superior lymph node of the CHA that had infiltrated the head of the pancreas and the CHA, and the tumor markers remained low; therefore, CS was performed. The postoperative histopathological evaluation showed no residual viable tumor cells in the primary tumor and the metastasis in the anterior superior lymph node of the CHA, and a pathological complete response was confirmed.

In the present case, the patient received 6-month adjuvant pembrolizumab monotherapy. The recommended regimen under the Japanese Gastric Cancer Treatment guidelines 2021 encompasses 1-year S-1 monotherapy for stage II and combination therapy (e.g., one-year S-1 plus docetaxel or 6-month capecitabine plus oxaliplatin) for stage III, whereas pembrolizumab is not recommended due to the absence of relevant clinical trial data. The decision to offer adjuvant pembrolizumab monotherapy, in this case, was based on previous reports demonstrating little efficacy of cytotoxic chemotherapy in MSI-high gastric cancer [16, 17]; and favorable results in clinical trials of this therapy for melanoma [22]. The patient’s complete pathological response also underscored this rationale. These points were discussed with the patient when presenting him with the option of adjuvant pembrolizumab monotherapy, and he decided to proceed with this non-standard option. We consider that adjuvant pembrolizumab monotherapy was effective in the present case but acknowledge that further evidence from clinical trials is needed before this therapy can be regarded as standard.

## Conclusions

Here, we describe the CS for MSI-high gastric cancer with a complete pathological response to pembrolizumab. We consider that this therapeutic approach has the potential to enhance outcomes for MSI-high gastric cancer patients.

## Data Availability

The datasets obtained during the current study are available from the corresponding author on reasonable request.

## Abbreviations

BSC: Best supportive care
CA: Carbohydrate antigen
CEA: Carcinoembryonic antigen
CHA: Common hepatic artery
CS: Conversion surgery
CT: Computed tomography
dMMR: Mismatch repair deficiency
EGD: Esophagogastroduodenoscopy
HER2: Human epidermal growth factor receptor 2
ICI: Immune checkpoint inhibitor
MSI: Microsatellite instability
MSS: Microsatellite stable
MST: Median survival time
ORR: Objective response rate
OS: Overall survival
PD-1: Programmed death 1
PD-L1: Programmed death-ligand 1
RECIST: Response Evaluation Criteria in Solid Tumors
